# Prevalence and risk factors of food insecurity among Libyan migrant families in Australia

**DOI:** 10.1186/s12889-021-12202-9

**Published:** 2021-11-24

**Authors:** Reima Mansour, James Rufus John, Pranee Liamputtong, Amit Arora

**Affiliations:** 1 School of Health Sciences, Western Sydney University, Campbelltown Campus, Locked Bag 1797, Penrith, NSW 2751 Australia; 2 Health Equity Laboratory, Campbelltown, NSW 2560 Australia; 3 Department of Nutrition, Faculty of Public Health, Benghazi University, Benghazi, Libya; 4 School of Psychiatry, University of New South Wales, Sydney, NSW 2052 Australia; 5 South Western Sydney Local Health District, Liverpool, NSW 2170 Australia; 6 Ingham Institute of Applied Medical Research, Liverpool, NSW 2170 Australia; 7 Present Address: College of Health Sciences, VinUniversity, Gia Lam District, Hanoi, 100000 Vietnam; 8 Translational Health Research Institute, Western Sydney University, Locked Bag 1797, Penrith, NSW 2751 Australia; 9 Clinical School Child and Adolescent Health, The Children’s Hospital at Westmead Clinical School, Faculty of Medicine and Health, The University of Sydney, Westmead, NSW 2145 Australia; 10 Oral Health Services, Sydney Local Health District and Sydney Dental Hospital, NSW Health, Surry Hills, NSW 2010 Australia

**Keywords:** Food insecurity, Migrants, Refugees, Socio-economic inequality, Social correlates of health, Australia

## Abstract

**Background:**

The burden of food insecurity remains a public health challenge even in high income countries, such as Australia, and especially among culturally and linguistically diverse (CALD) communities. While research has been undertaken among several migrant communities in Australia, there is a knowledge gap about food security within some ethnic minorities such as migrants from the Middle East and North Africa (MENA). This study aims to determine the prevalence and correlates of food insecurity among Libyan migrant families in Australia.

**Methods:**

A cross-sectional design utilising an online survey and convenience sampling was used to recruit 271 participants, each representing a family, who had migrated from Libya to Australia. Food security was measured using the single-item measure taken from the Australian Health Survey (AHS) and the 18-item measure from the United States Department of Agriculture Household Food Security Survey Module (USDA HFSSM). Multivariable logistic regression was used to identify independent correlates associated with food insecurity.

**Results:**

Using the single-item measure, the prevalence of food insecurity was 13.7% whereas when the 18-item questionnaire was used, more than three out of five families (72.3%) reported being food insecure. In the multivariable logistic regression analysis for the single-item measure, those living alone or with others reported higher odds of being food insecure (AOR = 2.55, 95% CI 1.05, 6.21) compared to those living with their spouse, whereas higher annual income (≥AUD 40,000) was associated with lower odds of food insecurity (AOR = 0.30, 95% CI 0.11, 0.84). Higher annual income was also associated with lower odds of food insecurity (AOR = 0.49, 95% CI 0.25, 0.94) on the 18-item measure. On both single and 18-item measures, larger family size (AOR = 1.27, 95% CI 1.07, 1.49 and AOR = 1.21, 95% CI 1.01, 1.47 respectively) was associated with increased odds of food insecurity.

**Conclusion:**

This study provides evidence that food insecurity amongst Libyan migrants in Australia is a widespread problem and is associated with a number of sociodemographic and socio-economic factors. The findings of this study serve to contribute to the depth and breadth of food security research among vulnerable communities, in this instance Libyan migrant families.

**Supplementary Information:**

The online version contains supplementary material available at 10.1186/s12889-021-12202-9.

## Background

Food security exists when all people, at all times, have physical and economic access to sufficient, safe and nutritious food that meets their dietary needs and food preferences for an active and healthy life [1, 2], physically and mentally [3, 4]. Contrarily, food insecurity refers to limited access to food, at the level of individuals or households, due to lack of money or other resources [5]. Long-term effects of food insecurity can include increased prevalence of serious chronic illnesses [6, 7], poor skeletal growth, asthma, and poor mental health [8, 9]. Globally, the burden of food insecurity remains a challenge and has consistently increased at the global level since 2014 [5]. According to the current Food and Agriculture Organization report, the global prevalence of food insecurity is estimated to be 25.9% (2 billion people) in 2019 [5]. Preliminary assessments in 2020 suggest that the COVID-19 pandemic might have increased the total number of undernourished and food insecure people worldwide [5]. Hunger and poverty are significant issues linked with low- and middle-income countries; however, food insecurity is also prevalent among certain minority groups in high income countries [10, 11]. Zero Hunger is the second of the United Nation’s Sustainable Development Goals [12]. Meeting this goal involves meeting the needs of vulnerable populations such as migrants and refugees; this being so, research among these populations is crucial.

In Australia, the prevalence of food insecurity among the general population was approximately 4% in 2011/12, according to the Australian Health Survey (AHS) [13]. Although overall food insecurity is low, the latest AHS estimated that 3.5% of households in the most populous state, New South Wales (NSW), had experienced food insecurity as they had reported not having sufficient food to eat [13] at some time during the previous year, while a recent systematic review estimated the prevalence of food insecurity ranged from 2 to 90% among various population groups in Australia [14]. A recent study has found that since the onset of the COVID-19 pandemic, approximately 26% Australians (1 in 4) have experienced food insecurity to some degree [15].

Food insecurity is inextricably linked to sociodemographic and socioeconomic characteristics that influence food equity and accessibility among different population groups [16, 17]. Hence, some population groups are significantly affected, including young people, single people living alone, single parent families, the unemployed [18], the elderly, low-income earners [2, 19] (including those with limited and/or insecure employment [19, 20]), welfare recipients [2, 21], those with a disability [22] and large households [23]. In addition, almost one in ten people reported avoiding particular foods for cultural, religious, or ethical reasons [24]. This sheds light on the existence of potential dimensions other than affordability and availability as food security factors.

People from culturally and linguistically diverse (CALD) backgrounds have been identified as vulnerable to food insecurity [19]. This applies particularly to refugees and migrants, where food insecurity has been identified as being as high as 90% [25, 26]. According to a study among migrants in urban and regional Tasmania, 9% have gone without food at some point; the main reasons were: food being too expensive, shortage of funds, unavailability of desired food, and the distance to shops (>4kms) [27]. Several factors may overlap or intersect for both refugees and migrant families, including precarious employment, larger family size, and cultural and linguistic differences to the dominant culture combined with trauma, dislocation, familial separation, and educational disadvantage [23, 27]. Food insecurity commonly occurs in specific disadvantaged areas where CALD communities and/or low income populations are concentrated [19, 27]. Little research, however, has been done on recent migrant and/or refugee groups.

Although there is evidence on food security status among some established migrant populations, there is a knowledge gap and limited understanding of food security among some more recently arrived migrants such as those from the Middle East and North Africa (MENA) among whom little research has been undertaken [28, 29]. In a recent systematic review, only three studies, all from the United States [8, 30, 31], reported data on MENA migrants; they found varying insecurity prevalence, namely 40% [8], 60% [30] and 71% [31]. However, no study to date has specifically explored food security status among Libyan migrants. Therefore, this study aims to address the knowledge gap in determining the prevalence of and correlates associated with food insecurity among Libyan migrant families currently living in Australia.

## Methods

### Study design, participants, and recruitment

The study adopted a cross-sectional design utilising an online survey to investigate the prevalence of food insecurity among Libyan families in Australia. Participants were drawn from an estimated population of 500 families (comprised of a total of about 2810 individuals) [32] with the majority in NSW [33].

Participants were initially informed about the project in a study invitation letter distributed via the Australian Libyan Association Incorporation and the Libyan Embassy in Australia. The ‘snowball’ method of participant recruitment was chosen as an effective way to access families across Australia, as it increased the potential to maximise the sample size [34, 35]. The survey used to gather data for this study was sent via email through the Libyan Embassy, and the Australian Libyan Association Incorporation offices. To broaden the opportunity to include participation by those not in contact with the Embassy and to further foster a ‘snowballing sampling’ [36], a link to the survey was placed on the social media presences of Libyan immigrant groups (on Facebook, Instagram, and WhatsApp). The recruitment process commenced October 2019, with four reminders sent in the period October 2019 to February 2020. The survey included a statement indicating that the participants implied their consent by accessing and completing the online survey.

### Sampling

Convenience sampling (a non-probability sampling strategy), as outlined above, was used to recruit Libyan migrants in Australia as it is time- and cost-effective manner of accessing a target population [37]. A sample size calculation was adopted to estimate the minimum number of respondents required for the results to have sufficient statistical power. The minimum sample size required to ensure a maximum margin of error of ±5% was 235 families. This calculation was based on a total population [38] of approximately 500 families, with a margin of error of 5%, assuming a 50% response rate (as this gives the maximum possible sample size) and a confidence level of 95% [39, 40].

### Data collection

The survey included questions regarding basic socio-demographic and socio-economic factors such as age, education, and income. Participants were also asked for information about their food experiences, specifically food access, availability, level of food security since they started living in Australia. The survey took approximately 30 min to complete. It should also be noted that because the online survey was anonymous (rather than reliant on later de-identification of material), it was impossible for data to be withdrawn once the fully completed survey had been submitted. Hence, there are no participant withdrawals subsequent to data collection. As data from incomplete surveys were discarded, all analysed data were from fully-completed surveys.

The questionnaire used in this study (see Additional file 1) was developed based on two measures of food insecurity previously used in large scale research in countries including the USA, Canada, and Australia [11, 26, 31, 41]. The first measure was the US Household Food Security Survey Module (USDA HFSSM) which is an 18-item scale used for households with children. It is a comprehensive and validated tool for measuring food insecurity [42] and was derived from the US Department of Agriculture (USDA) Community Food Security Assessment Toolkit. The second measure was a single-item measure taken from the AHS to identify food insecurity: “In the last 12 months was there any time you have run out of food and not been able to purchase more?” [43]. The single item is included in this study, so the results of this study can be compared with the results from the general population. By using these two popular measures study attempted to explore both measures and determine which captured more information or factors related to food insecurity and the extent of any such association. The online version of the survey was designed and data was collected via *Qualtrics* (online survey software, Provo, UT, USA) [44].

### Covariates

Socio-demographic and socio-economic data were collected from participants. Socio-demographic data collected comprises their age (in years), gender (M/F), length of stay (years), citizenship/visa type, English language proficiency (low, intermediate, high), State of residence (NSW, Victoria, Queensland, others), education (Year 10 or less, completed high school, vocational, undergraduate university, postgraduate university), household structure (living with spouse, living with others), number of family members, and number of children in family. Socio-economic attributes included employment status (yes/no), annual income (<AUD40,000/≥AUD40,000), occupational status (managerial, professionals or skilled/ unskilled, pensioner or unemployed), private health insurance status (yes/no), housing arrangements (renting, paying-off mortgage, outright owner / fully owned), and location and its socio-economic status (SES) as indicated by residential postcode as per the Index of Relative Socioeconomic Advantage and Disadvantage (IRSAD) [45].

### Statistical analysis

Descriptive data analysis included the calculation of the overall prevalence of food insecurity, stratified by gender and age. Continuous variables were shown as means and standard deviation (SD) whereas categorical variables were shown as percentages. Chi-square tests were used to test for significant differences between categorical variables, i.e., various socio-demographic (e.g., gender, language proficiency) and socio-economic factors (e.g., income, employment status) and food insecurity. In terms of the continuous variables (such as age, number of people in a household), independent samples t-tests were used to test for differences in the means of those variables between those respondents who were food secure and those who were food insecure.

The outcome variables using the single-item measure of food insecurity was a dichotomous response (yes/no). Binary coding was used for responses to the USDA 18-item food security questions which were first coded as food secure or insecure (scored 0 and 1, respectively). They were then combined into a single overall measure called the “food security scale”. The sum of the scores determined the categorisation of the degree of severity of food insecurity/hunger into 3 categories: (1) food secure (where the sum of scores was 0–2); (2) food insecure without hunger (where sum of the scores was 3–7); (3) food secure with hunger (where the sum of the scores was 8–18) [1].

Univariable logistic models quantified the unadjusted associations between various factors and food insecurity. Subsequently, multivariable regression analysis was used to determine the association between the socio-demographic and socio-economic variables and the two main outcome variables. All explanatory variables were entered into the full model which was reduced using the backward stepwise procedure (p for removal < 0.05) and the fitness of model was assessed at every step to avoid dropping non-significant variables that affected the model fitness. The backward stepwise regression procedure was used to develop models to predict food insecurity correlates with adjusted odds ratios (AOR) and 95% confidence intervals. Additionally, a Pearson’s correlation coefficient test was used to investigate any significant collinearity among variables in the models and eliminate any variable that demonstrated strong collinearity with each other. A significance or Alpha (α) level of 0.05 was used for all analyses. Finally, variables that had significant statistical association (*p* < 0.05) with food insecurity remained in the final model. Version 26 of IBM’s Statistical Package for the Social Sciences (SPSS) software was used (Armonk, NY: IBM Corp.).

## Results

### Baseline characteristics

The demographic characteristics of the study population are shown in Table 1. Of the estimated 500 Libyan migrant families in Australia, the survey reached at least 303 families residing in seven States and Territories of Australia who began the online questionnaire, of whom 32 failed to complete their surveys. The survey was designed so that respondents were required to complete each question in order to progress to the next. Only full completed surveys were included in the analyses. Failure to complete a fully completed survey resulted in data from that survey not being used. Thus, information on all variables was complete for all participants whose data was used. Thus, 271 (54.2%) fully completed the survey and were included in the final analysis.

**Table 1:** Food security status of the sample using single-item and 18-item measures in relation to the demographic attributes and socioeconomic factors

Factors	Total N (%)	Food insecurity measure %		Single item ***p***-value	18-item ***p***-value
		FI, single item	FI, 18-item		
		N (%) or mean (SD)	N (%) or mean (SD)		
**Socio-demographic attributes**					
**Age, Mean (SD)**	38.2 (6.9)	38.6 (5.8)	38.7 (6.6)	0.648	0.041
**Age in categories**					
20–29 years	37 (13.7)	3 (8.10)	21 (10.7)		
30–39 years	113 (40.2)	15 (40.5)	77 (39.3)	0.241	0.010
40–49 years	109 (41.7)	19 (51.4)	90 (45.9)		
50 years and above	12 (4.4)	0 (0.0)	8 (4.1)		
**Gender**				0.615	0.200
Male	107 (39.5)	16 (43.2)	82 (41.8)		
Female	164 (60.5)	21 (56.8)	114 (58.2)		
**Length of stay, Mean (SD)**	8.8 (2.7)	9.03 (2.9)	8.45 (3.0)	0.532	0.247
1–5 years	32 (11.9)	3 (8.1)	20 (10.2)		
6–10 years	203 (75.2)	30 (81.1)	150 (76.5)	0.651	0.394
11–15 years	35 (13.0)	4 (10.8)	26 (13.3)		
**Citizenship/visa type**				0.463	0.129
Australian citizen	181 (66.8)	26 (70.3)	132 (67.3)		
Permanent residence	68 (25.1)	10 (27.0)	52 (26.5)		
Temporary visa	22 (8.1)	1 (2.7)	12 (6.1)		
**English language proficiency**				0.916	0.524
Low level	56 (20.7)	7 (18.9)	38 (19.4)		
Intermediate level	77 (28.4)	10 (27.0)	59 (30.1)		
High level	138 (50.9)	20 (54.1)	99 (50.5)		
**State of residence**				0.463	0.113
NSW	150 (55.4)	24 (64.9)	102 (52)		
Vic	58 (21.4)	7 (18.9)	46 (23.5)		
Qld	29 (10.7)	4 (10.8)	25 (12.8)		
Others	34 (12.5)	2 (5.4)	23 (11.7)		
**Education**				0.182	0.416
Vocational, high school or less	45 (16.6)	10 (27.0)	36 (18.4)		
Undergraduate University	71 (26.2)	9 (24.3)	49 (25.0)		
Postgraduate University	155 (57.2)	18 (48.6)	111 (56.6)		
**Household structure**				0.006	0.781
Living with spouse	236 (87.1)	27 (73.0)	170 (86.7)		
Living with others	35 (12.9)	10 (27.0)	26 (13.3)		
**Number of family members, Mean (SD)**	5 (1.6)	4.6 (2.0)	5.2 (1.7)	0.123	0.004
2–5 people	154 (56.8)	25 (67.6)	99 (50.5)		
6–10 people	117 (43.2)	12 (32.4)	97 (49.5)	0.156	0.001
**Number of children in families, Mean (SD)**	2.9 (1.6)	3.05 (2.0)	3.17 (1.5)	0.612	0.000
0	35 (12.9)	6 (16.2)	19 (9.7)		
1–2	56 (20.7)	8 (21.6)	37 (18.9)		
3–4	145 (53.5)	14 (37.8)	109 (55.6)	0.830	0.007
5 or more	35 (12.9)	9 (24.3)	31 (15.8)		
**Socio-economic attributes**					
**Employment status**				0.430	0.988
Yes	101 (37.4)	16 (43.2)	73 (37.4)		
No	169 (62.6)	21 (56.8)	122 (62.6)		
**Annual income**				0.003	0.052
<AUD40,000	139 (64.4)	31 (86.1)	112 (67.9)		
≥AUD40,000	77 (35.6)	5 (13.9)	53 (32.1)		
**Occupation status**^a^				0.845	0.969
Managerial, professionals or skilled	55 (21.1)	8 (22.2)	40 (21.1)		
Unskilled, pensioner or unemployed	207 (79)	28 (77.8)	150 (78.9)		
**Private health insurance**				0.245	0.985
Yes	54 (19.9)	10 (27.0)	39 (19.9)		
No	217 (80.1)	27 (73.0)	157 (80.1)		
**Housing arrangements**				0.077	0.782
Renting	260 (95.9)	33 (89.2)	189 (96.4)		
Paying-off mortgage	5 (1.8)	2 (5.4)	3 (1.5)		
Outright owner or fully-owned	6 (2.2)	2 (5.4)	4 (2.0)		
**IRSAD**^b^				0.889	0.395
1–2	31 (11.6)	3 (8.3)	19 (9.7)		
3–4	56 (21.0)	8 (22.2)	40 (20.5)		
5–6	40 (15.0)	4 (11.1)	33 (16.9)		
7–8	102 (38.2)	15 (41.7)	75 (38.5)		
9–10	38 (14.2)	6 (16.7)	28 (14.4)		

^a^Open ended question

^b^ Index of Relative Socio-economic Advantage and Disadvantage, Postal Area Code (POA) (Ranking within Australia, Socio-Economic Indexes for Areas (SEIFA))

Note: Three postcodes were not found on the 2016 ABS SEIFA (2186, 2610, 3336). Our data was collected at the end of 2019 and the beginning of 2020 so these may be new suburbs

The mean age of the respondents was 38 ± 7 years with a predominance of female respondents (89.5%). Most respondents (87%) had children, with 53.5% of them having 3 to 4 children. Families ranged in size from 2 to 10 members, with an average size of 5. A predominant percentage of participants reported residing in NSW (55%) followed by Victoria (21%). In terms of education, 57% reported having a postgraduate university degree. However, 63% reported being unemployed. In terms of Index of Relative Socioeconomic Advantage and Disadvantage, the majority of interviewees (*n* = 102, 83%) fell within deciles 7 & 8 (highly advantaged/low disadvantaged). A further 96 families (36%) fell in deciles 3 & 4 and 5 & 6 (highly disadvantaged and moderately disadvantaged, respectively).

In the single-item measure, chi-squared tests showed statistically significant differences in the relationship of household structure (living with spouse (73%) and of income levels between those who were food secure and those who were food insecure. In terms of income, the single-item measure found that 86% of families earning less than AUD 40,000 per annum reported being food insecure; while a slightly lower response was obtained using the 18-item measure, with 67.9% of respondents earning less than AUD 40,000 reporting being food insecure (see Table 1, from which is derived all the material in this section).

Using the 18-item measure, additional characteristics were associated with food insecurity, including age, number of children and family size. A higher proportion (about 50%) of older adults aged 40–49 years compared with younger adults were food insecure. With respect to family size, on average food insecure families had a greater number of people per household (M = 5.2, SD = 1.7) than food secure families. This difference was statistically significant. Additionally, food insecure families had more children (M = 3.17, SD = 1.5) than food secure families. This difference was significant and represented a medium to large effect.

### Prevalence of food insecurity using the single- and 18-item measures

Table 2 presents the prevalence and severity of food insecurity of the total study. Overall, the level of reported food insecurity using the single-item measure was 13.7% (*n* = 37), whereas 234 families were food secure. However, using the 18-item measure, more than three in five families (72.3%, *n* = 196) reported having at least 3 indicators of food insecurity. Larger households with children were associated with food insecurity and with increased severity compared to households without children. With respect to families with children, 65.3% (*n* = 177) of such families were food insecure using the 18-item measure, while the figure was lower for the single-item measure, namely 11.4% (*n* = 31). In terms of the severity of food insecurity, the 18-item measure found 64.2% (*n* = 174) were food insecure without hunger, compared to 8.1% (*n* = 22) families who suffered food insecurity with hunger. In terms of families with children, 58.7% of such families suffered food insecurity without hunger while 6.6% suffered food insecurity with hunger. For families without children, 5.5% suffered food insecurity without hunger and 1.5% suffered food insecurity with hunger.

**Table 2:** Prevalence of food insecurity among Libyan families in Australia

Factors	Total (n)	Food insecurity			
		Single item n (%)	18-item n (%)		The full 18-item results n (%)
			Without hunger	With hunger	
**Total families**	271	37 (13.7)	174 (64.2)	22 (8.1)	**196 (72.3)**
**Households with children**	236	31 (11.4)	159 (58.7)	18 (6.6)	**177 (65.3)**
**Households without children**	35	6 (2.2)	15 (5.5)	4 (1.5)	**19 (7.0)**

### Univariable analysis

Univariable analysis for the single-item measure of food insecurity (Table 3) demonstrated that single parent families and multi-family households had about three times higher risk of experiencing food insecurity (OR = 3.09; 95% CI 1.34, 7.14) than two parent families. Additionally, annual income and household structure were associated with food insecurity. Families on higher annual income (AUD 40,000 or above) were 4 times more food secure than those on incomes below that Fig. (OR = 4.13; 95% CI 1.53, 11.1).

**Table 3:** Univariable and multivariable logistic regression for single-item food insecurity measure – Odds Ratio (OR) and 95% Confidence Interval (CI)

Variable	Univariable single-item N (%)			Multivariable single-item N (%)		
	OR	95% CI	p-value	AOR	95% CI	*p-*value
**Socio-demographics attributes**						
**Age**	0.99	(0.94, 1.04)	0.646	Non-significant in final model		
**Gender**						
Male	Reference category			Insignificant in final model		
Female	1.20	(0.59, 2.41)	0.615			
**Length of stay**	0.96	(0.85, 1.09)	0.530	Non-significant in final model		
**Citizenship/visa type**						
Australian citizen	Reference category			Non-significant in final model		
Permanent residence	0.97	(0.44, 2.14)	0.946			
Temporary visa	3.52	(0.45, 27.33)	0.228			
**English language proficiency**						
Low level	Reference category			Non-significant in final model		
Intermediate level	0.96	(0.34, 2.69)	0.934			
High level	0.84	(0.33, 2.12)	0.717			
**State of residence**						
NSW	Reference category			Non-significant in final model		
Vic	1.39	(0.56, 3.42)	0.477			
Qld	1.19	(0.38, 3.73)	0.765			
Others	3.05	(0.68, 13.60)	0.144			
**Education**						
Vocational, high school or less	Reference category			Non-significant in final model		
Undergraduate University	1.97	(0.73, 5.30)	0.181			
Postgraduate University	2.18	(0.92, 5.12)	0.076			
**Household structure**						
Living with spouse	Reference category					
Living with others	3.09	(1.34, 7.14)	0.008	2.56	(1.05, 6.21)	0.039
**Number of family members**	0.82	(0.66, 1.01)	0.065	1.27	(1.07, 1.49)	0.005
**Number of Children among families**						
0	Reference category			Non-significant in final model		
1–2	1.24	(0.39, 3.94)	0.714			
3–4	1.94	(0.69, 5.46)	0.212			
5 or more	0.60	(0.19, 1.91)	0.385			
**Socio-economic attributes**						
**Employment status**						
Working	Reference category			Non-significant in final model		
Not working	1.33	(0.66, 2.68)	0.431			
**Annual income**						
<AUD40,000	Reference category					
≥AUD40,000	4.13	(1.53, 11.10)	0.005	0.30	(0.11, 0.84)	0.022
**Occupation status**^a^						
Managerial, professionals or skilled	Reference category			Non-significant in final model		
Unskilled, pensioner or unemployed	0.92	(0.39, 2.1)	0.845			
**Private health insurance**						
Yes	Reference category			Non-significant in final model		
No	1.60	(0.72, 3.55)	0.248			
**Housing arrangements**						
Renting	Reference category			Non-significant in final model		
Paying-off mortgage	0.22	(0.035, 1.35)	0.102			
Outright owner or fully-owned	0.29	(0.051, 1.65)	0.163			
**IRSAD**^b^						
1–2	Reference category			Non-significant in final model		
3–4	0.64	(0.15, 2.62)	0.538			
5–6	0.96	(0.19, 4.66)	0.964			
7–8	0.62	(0.17, 2.30)	0.477			
9–10	0.57	(0.13, 2.50)	0.457			

^a^ Open ended question

^b^ Index of Relative Socio-economic Advantage and Disadvantage, Postal Area Code (POA) (Ranking within Australia, Socio-Economic Indexes for Areas (SEIFA))

In relation to the 18-item measure (Table 4), with every increase in age of the respondent (with age ranging from 20 to 57) by a year, there was a 4% increase in the odds of being food insecure (OR = 1.04; 95% CI 1.01, 1.08). Moreover, large families had 27% increased risk of being food insecure (OR = 1.27; 95% CI 1.07, 1.49) compared to small families. Correspondingly, families with 5 or more children were significantly associated with increased risk of food insecurity (OR = 6.53; 95% CI 1.90, 22.45) compared to families who had fewer or no children.

**Table 4:** Univariable and multivariable logistic regression for the 18-item food insecurity measure – Odds Ratio (OR) and 95% Confidence Interval (CI)

Parameter	Univariable 18-item N (%)			Multivariable 18-item N (%)		
	OR	95% CI	p-value	AOR	95% CI	p-value
**Socio-demographics attributes**						
**Age**	1.04	(1.01,1.08)	0.043	Non-significant in final model		
**Gender**						
Male	Reference category			Non-significant in final model		
Female	0.70	(0.40, 1.21)	0.201			
**Length of stay**	1.06	(0.96, 1.17)	0.247	Non-significant in final model		
**Citizenship/visa type**						
Australian citizen	Reference category			Non-significant in final model		
Permanent residence	1.21	(0.63, 2.31)	0.571			
Temporary visa	0.45	(0.18, 1.10)	0.079			
**English language proficiency**						
Low level	Reference category			Non-significant in final model		
Intermediate level	1.55	(0.72, 3.35)	0.263			
High level	1.20	(0.61, 2.35)	0.591			
**State of residence**						
NSW	Reference category			Non-significant in final model		
Vic	1.80	(0.88, 3.71)	0.109			
Qld	2.94	(0.97, 8.92)	0.057			
Others	0.98	(0.44, 2.22)	0.968			
**Education**						
Vocational, high school or less	Reference category					
Undergraduate University	0.56	(0.23, 1.35)	0.196	Non-significant in final model		
Postgraduate University	0.63	(0.28, 1.42)	0.264			
**Household structure**						
Living with spouse	Reference category			Non-significant in final model		
Living with others	1.12	(0.59, 2.52)	0.781			
**Number of family members**	1.27	(1.07, 1.49)	0.005	1.21	(1.01, 1.47)	0.048
**Number of Children among families**						
0	Reference category			Non-significant in final model		
1–2	1.64	(0.69, 3.90)	0.262			
3–4	2.55	(1.19, 5.50)	0.016			
5 or more	6.53	(1.90, 22.45)	0.003			
**Socio-economic attributes**						
**Employment status**						
Employed	Reference category			Non-significant in final model		
Unemployed	0.99	(0.57, 1.73)	0.988			
**Annual income**						
< AUD40,000	Reference category					
≥AUD40,000	0.53	(0.28, 1.01)	0.053	0.49	(0.25, 0.94)	0.032
**Occupation status**^a^						
Managerial, professionals or skilled	Reference category					
Unskilled, pensioner or unemployed	0.99	(0.51,1.92)	0.969	Non-significant in final model		
**Private health insurance**						
Yes	Reference category			Non-significant in final model		
No	1.01	(0.52, 1.96)	0.985			
**Housing arrangements**						
Renting	Reference category			Non-significant in final model		
Paying-off mortgage	0.56	(0.09, 3.44)	0.534			
Outright owner or fully-owned	0.75	(0.13, 4.19)	0.744			
**IRSAD** ^b^						
1–2	Reference category			Non-significant in final model		
3–4	1.58	(0.62, 3.99)	0.334			
5–6	2.98	(1.01, 8.85)	0.050			
7–8	1.75	(0.75, 4.09)	0.193			
9–10	1.77	(0.64, 4.91)	0.274			

^a^ Open ended question

^b^ Index of Relative Socio-economic Advantage and Disadvantage, 2016 Postal Area Code (POA) (Ranking within Australia, Socio-Economic Indexes for Areas (SEIFA)

### Multivariable analysis

The multivariable models for single and 18-item measures are shown in Tables 3 and 4, respectively. For the single item measure, in terms of household structure, respondents who reported living as a single parent or in a multi-family household had more than two times higher odds of food insecurity (AOR = 2.55; 95% CI 1.05, 6.21) than respondents living with a spouse or partner. Additionally, large families were associated with 27% higher odds of food insecurity (AOR = 1.27, 95% CI 1.07, 1.49). Income was a significant predictor of food insecurity where families with high income were associated with 70% lower odds of food insecurity (AOR = 0.30; 95% CI 0.11, 0.84) when compared with lower income families.

The multivariable analysis for the 18-item measure (Table 4) showed that large households had 21% increased odds of being food insecure (AOR = 1.21, 95% CI 1.01, 1.47) while smaller households tended to be more food secure. In terms annual income, families with high annual incomes (<AUD 40,000) had 50% lower odds of food insecurity (AOR 0.49, 95% CI 0.25, 0.94). Both the single and 18-item measures revealed that lower income and greater family size are significantly associated with food insecurity. Other variables (including age, length of stay and education) were non-significant in the final model (see Table 4).

## Discussion

The present study used both single-item measure of food insecurity used previously in the AHS and the 18-item measure of food insecurity developed in the USA and used in international and Australian studies. We found that Libyan migrants in Australia experienced higher than normal levels of food insecurity reported among the general adult Australian population using the single item measure. The level of food insecurity (at 13.7%) was about three times more than that observed (4%) in the last national general survey of food insecurity in Australia in 2005 [43] which used the same measure. Using the 18-item measure of food insecurity, the current study found a prevalence of 72.3%. This is even higher than that found in a recent study that described the prevalence of food insecurity in Tasmania (Australia) during the COVID-19 pandemic [15]. Using the USDA measure (Short Form), Kent et al. (2020) found a general Australian food insecurity prevalence rate of 26% [15]. The level of food insecurity found in this study was consistent with those found in other studies among migrant and refugee populations in Australia [25, 26, 46], the USA [8, 30, 31, 47] and other high-income countries [48–51].

Findings from the multivariable regression analysis using both single and 18-item measures consistently showed that family size and annual income were significant correlates of food insecurity among Libyan migrants in Australia. In this study, in relation to household structure and size, on average, both larger families and single parents were more food insecure than couples with or without children. This echoes findings in other Australian and international research on food insecurity [3, 23, 52–54]. In contrast to these findings, other studies have revealed that food insecurity status is negatively related to family size [7, 10]; however, this was associated with households receiving additional support through welfare vouchers. A study among asylum seekers living in Norway found that households with more children were associated with low food insecurity status due to additional government assistance being provided for larger families [48]. This indicates that family size and income impacts may be hypothesised to be lessened by government policies and intervention.

Income was one of the significant correlates of food insecurity. Families on an annual income of less than AUD 40,000 were twice as likely to report that they were food insecure than those who were earning more. This research is consistent with the findings of Kent et al. [15] and others [23, 52, 55] on the relationship between higher income and greater food security.

In another recent US research study, food insecurity associated with unemployment was reduced where unemployed people received proceeds of unemployment insurance [56]. In Australia, unemployment insurance uptake remains lower than might be otherwise expected, however, due to the availability of unemployment benefits (although broadly criticised for their inadequacy) and the impact of receiving accumulated leave entitlements [57]. This study demonstrates that employment, while a protective factor against food insecurity, did not eliminate it.

### Strengths, limitations, and future directions

It is the first study of its kind for this minority population, namely Libyan migrants in Australia. Its strengths include a comprehensive exploration of the correlates of food insecurity among a little studied comparatively recently arrived population in Australia, and a population whose primary language is other than English. The Libyan families in this study were also geographically located across various Australian states, thereby providing diverse perspectives and insights into food security research. The research team partnered with the Libyan embassy and simultaneously utilised social media platforms such as (Facebook, Instagram, and WhatsApp) to enhance participant recruitment. We also used validated single-item and 18-item food security measures for national and international comparisons.

Nevertheless, there are several limitations of this study. While the response rate was high (over 50%), generalisability may be limited due to self-selection bias and the possibility that the study sample may not be fully representative of Libyan migrant population in Australia. The study findings are also limited to Libyan families in Australia and may not represent the experiences of other migrant populations. Future research should consider exploring food insecurity among other recent migrant populations in Australia and overseas. Another limitation is that the distribution among subgroups within the survey sample may be unevenly distributed. For example, some data such as house ownership responses were skewed towards rental accommodation. Nonetheless, as rental accommodation is often common among recent migrants than longer term migrants [58] (perhaps due to the high cost of home ownership in Australia), the skew may be representative of that distribution in the population. Some results generated large confidence interval which could indicate an input variable of dubious merit or identify spurious associations. This is a risk where a multivariable logistic regression model has been constructed with many variables that genuine associations may be diluted [59].

There is abundant room for further investigation on food insecurity and provision of support among migrants who are likely to be at high risk of food insecurity. Data from this study indicates that the single-item measure underestimated the extent of the problem; however, both the single and the 18-item measure did not include reference to factors such as limited transport access, special food needs and cultural food preferences. Food security dimensions are broader than financial factors. It is therefore recommended that future studies include random sampling for increased generalisability and the use of collaborative methodologies that would explore all the dimensions and complexities of food insecurity among migrants.

## Conclusion

This is the first study to explore the prevalence and correlates of food insecurity amongst Libyan migrants. This study provides evidence that food insecurity amongst Libyan migrants in Australia is a widespread problem and is associated with significant sociodemographic and socio-economic factors including larger family size, low income, and single parent household. Despite the cross-sectional snapshot highlighted by this research, our findings serve to contribute to the depth and breadth of food security research among vulnerable communities, in particular Libyan migrant families in Australia. The study findings also highlight the need for further research to enable the provision of well-targeted support to alleviate the burden of food insecurity among migrant communities. Such research may also assist the government meet the demands of the United Nations’ Sustainable Development Goal No. 2: Zero Hunger (especially Target 2.1).

## Supplementary Information

**Additional file 1.** Food security survey among Libyan migrant families in Australia. Questionnaire including sociodemographic, socioeconomic and food security questions.

## Data Availability

The datasets generated and/or analysed during the current study are not publicly available due to the ethics approval’s terms.

## Abbreviations

AHS: Australian Health survey
USDA HFSSM: US Department of Agriculture (Household Food Security Survey Module)
CALD: Culturally and linguistically diverse
MENA: Middle Eastern North Africa
COVID-19: CoronaVirus Disease 19
