# Supplementary material for: Prevalence and risk factors of food insecurity among Libyan migrant families in Australia
Source: BMC Public Health. 2021 Nov 24;21:2156. doi: 10.1186/s12889-021-12202-9 (PMC8611633; doi:10.1186/s12889-021-12202-9)
Supplement: Supplementary file 1 — Additional file 1. Food security survey among Libyan migrant families in Australia. Questionnaire including sociodemographic, socioeconomic and food security questions. [file 12889_2021_12202_MOESM1_ESM.docx]

**FOOD SECURITY SURVEY AMONG LIBYAN MIGRANT FAMILIES IN AUSTRALIA**

Q1- How old are you?

Q2- What is your gender?

- Male
- Female

Q3- How long have you been in Australia?

Q4- What is your post code?

Q5- Are you?

- Australian Citizen
- Permanent resident
- Other Australian visa classes. Please specify……………….

Q6- How well do you speak English?

- Extremely well
- Very well
- Moderately well
- Slightly well
- Not well at all

Q7- What is your education level?

- Less than year 10
- Completed year 12
- Vocational (e.g., TAFE)
- Under-graduate (e.g., bachelor’s degree)
- Post-graduate (e.g., Master / Diploma / PhD)
- Other (Please specify) ………….

Q8- Which of the following would best describe the people who live in your household?

- I live alone
- I live in group household, unrelated adults (house/flat mates)
- I live with my husband / wife
- I live with my parents / relatives
- Other (Please specify) ………….

Q9- How many people (adults and children), including yourself, usually live in your household?

Q 10- Do you have private health insurance?

- Yes
- No

Q11- In the last 12 months, or since you arrived in Australia, were there any times that you ran out of food and could not afford to buy more?

- Yes
- No

Q12- How often have the following statements been true for you in the last 12 months or since you arrived in Australia?

|  | Often | Sometimes | Never |
| --- | --- | --- | --- |
| 1. I worry whether my food will run out before I get money to buy more |  |  |  |
| 1. The food that I bought just didn’t last, and I didn’t have money to get more |  |  |  |
| 1. I couldn’t afford to eat balanced meals |  |  |  |

Q13- In the last 12 months, or since you arrived in Australia, did (you/or other adults in your household) ever cut the size of your meals or skip meals because there wasn't enough money for food?

- Yes
- No

Q14- [IF YES ABOVE] How often did this happen?

- Almost every month
- Some months but not every month
- Only 1 or 2 months

Q15- In the last 12 months, or since you arrived in Australia, did you ever eat less than you felt you should because there wasn't enough money to buy food?

- Yes
- No

Q16- In the last 12 months, or since you arrived in Australia, were you ever hungry but didn't eat because you couldn't afford enough food?

- Yes
- No

Q17- Did you ever not eat for a whole day because there wasn't enough money for food?

- Yes
- No

Q18- [IF YES TO ABOVE QUESTION] How often did this happen?

- Almost every month
- Some months but not every month
- Only 1 or 2 months

Q19- In the last 12 months, or since you arrived in Australia, did you lose weight because you did not have enough money for food?

- Yes
- No

Q20- Do you have children?

- Yes
- No

Q21- How many children under the age of 18 years usually live in your house?

Q22- “I gave only a FEW kinds of LOW-COST FOOD to my child/children because I had LIMITED MONEY to buy food.” Was this true for your family in the last 12 months, or since you arrived in Australia?

- Often true
- Sometimes true
- Never true

Q23- “I COULDN’T FEED my child/children a NUTRITIONALLY BALANCED MEAL, because I couldn’t afford that.” Was this true for your family in the last 12 months, or since you arrived in Australia?

- Often true
- Sometimes true
- Never true

Q24- In the last 12 months, or since you arrived in Australia, did your child/children EVER SKIP MEALS because there wasn't enough money for food?

- Yes
- No

Q25- [IF YES TO ABOVE QUESTION] How often did this happen?

- Almost every month
- Some months but not every month
- Only 1 or 2 months

Q26- “My child /children DID NOT EAT ENOUGH because I just couldn't afford enough food.” Was this true for your family in the last 12 months, or since you arrived in Australia?

- Often true
- Sometimes true
- Never true

Q27- In the last 12 months, or since you arrived in Australia, did you ever REDUCE the SIZE of your child/children's MEALS because there wasn't enough money for food?

- Yes
- No

Q28- In the last 12 months, or since you arrived in Australia, was your child/children) EVERY HUNGRY but you just couldn't afford more food?

- Yes
- No

Q39- In the last 12 months, or since you arrived in Australia, did your child/ children EVER NOT EAT A WHOLE DAY because there wasn't enough money for food?

- Yes
- No

Q30- Are you currently employed?

- Yes
- No

Q31-What is your current job?

Q32- What is your annual total family income?

- - $0 – $39,999
  - $40000 – $59,999
  - $60000 - $79,999
  - $80000 – 99,999
  - More than 100000
  - Other please specify …………
